# Effect of Atorvastatin on Angiogenesis-Related Genes VEGF-A, HGF and IGF-1 and the Modulation of PI3K/AKT/mTOR Transcripts in Bone-Marrow-Derived Mesenchymal Stem Cells

**DOI:** 10.3390/cimb45030150

**Published:** 2023-03-10

**Authors:** Adriana Adamičková, Nikola Chomaničová, Andrea Gažová, Juraj Maďarič, Zdenko Červenák, Simona Valášková, Matúš Adamička, Jan Kyselovic

**Affiliations:** 1 5th Department of Internal Medicine, Faculty of Medicine, Comenius University Bratislava, 813 72 Bratislava, Slovakia; 2 Institute of Pharmacology and Clinical Pharmacology, Faculty of Medicine, Comenius University Bratislava, 813 72 Bratislava, Slovakia; 3 Clinic of Angiology, Comenius University and National Institute of Cardiovascular Diseases, 833 48 Bratislava, Slovakia; 4 Institute of Medical Biology, Genetics and Clinical Genetics, Faculty of Medicine, Comenius University Bratislava, 813 72 Bratislava, Slovakia; 5 Department of Pharmacology and Toxicology, University of Veterinary Medicine and Pharmacy in Kosice, 041 81 Kosice, Slovakia

**Keywords:** bone-marrow-derived mesenchymal stem cells, statins, regenerative therapy, growth factors, paracrine factors

## Abstract

Stem cell transplantation represents a unique therapeutic tool in tissue engineering and regenerative medicine. However, it was shown that the post-injection survival of stem cells is poor, warranting a more comprehensive understanding of activated regenerative pathways. Numerous studies indicate that statins improve the therapeutic efficacy of stem cells in regenerative medicine. In the present study, we investigated the effect of the most widely prescribed statin, atorvastatin, on the characteristics and properties of bone-marrow-derived mesenchymal stem cells (BM-MSCs) cultured in vitro. We found that atorvastatin did not decrease the viability of BM-MSCs, nor did it change the expression of MSC cell surface markers. Atorvastatin upregulated the mRNA expression levels of *VEGF-A* and *HGF*, whereas the mRNA expression level of *IGF-1* was decreased. In addition, the PI3K/AKT signaling pathway was modulated by atorvastatin as indicated by the high mRNA expression levels of *PI3K* and *AKT*. Moreover, our data revealed the upregulation of *mTOR* mRNA levels; however, no change was observed in the BAX and BCL-2 transcripts. We propose that atorvastatin benefits BM-MSC treatment due to its ability to upregulate angiogenesis-related genes expression and transcripts of the PI3K/AKT/mTOR pathway.

## 1. Introduction

Statins are a class of drugs that have been extensively studied for their efficacy in the prevention and treatment of cardiovascular diseases [1,2]. Statins target and inhibit 3-hydroxy-3-methylglutaryl coenzyme A (HMG-CoA) reductase, the rate-limiting enzyme of the mevalonate pathway, reducing cholesterol synthesis and increasing the uptake of low-density lipoproteins [3]. Within the mevalonate pathway, statins have additional cholesterol-independent effects, which involve the reduced synthesis of two isoprenoid intermediates, namely farnesylpyrophospate and geranylgeranylpyrophospate. These isoprenoid intermediates are necessary for post-translation modifications of numerous proteins, including the prenylation of small GTP-binding proteins belonging to the family of Ras, Rho, Rap and Rab GTPases [4]. The inhibited synthesis of isoprenoid intermediates may be involved in some of the statins’ cholesterol-independent “pleiotropic” effects. Examples of pleiotropic effects include improving the endothelial function, immunological, anti-apoptotic and antioxidant effects and regenerative capacity [5,6]. The inhibition of the mevalonate pathway holds an essential regulatory role in mesenchymal stem cell (MSC) biology by several signaling pathways, including MSCs differentiation, proliferation, angiogenic potential, survival and others [7]. One of these statin-mediated mechanisms may be the impact on the function of Rho-kinases, which act as serine/threonine kinase AKT inhibitors [8]. The phosphatidylinositol 3-kinase (PI3K) and the downstream target AKT belong to a conserved family of signal transduction enzymes with roles in cell proliferation, transformation, paracrine function and processes of angiogenesis and apoptosis [9]. Therefore, an understanding of the mechanism of the activation or modulation of PI3K/AKT pathway by statins in MSCs culture is warranted, as we already discussed in the review article [10]. In addition, statins can affect the expression of several growth factors, including vascular endothelial growth factor (VEGF), basic fibroblast growth factor (bFGF), insulin-like growth factor (IGF) and hepatocyte growth factor (HGF), which play important roles in the activation of the PI3K/AKT pathway. Multiple studies suggest that the combined use of statins and stem cell transplantation might improve the therapeutic efficacy of stem cells in regenerative medicine [11,12,13]. Among statins, atorvastatin represents the most widely prescribed statin worldwide, with many beneficial properties [14]. The objective of the present study was to evaluate the effect of atorvastatin on the characteristics and properties of bone-marrow-derived mesenchymal stem cells (BM-MSCs); mainly if atorvastatin affected the gene expression of BM-MSCs.

## 2. Materials and Methods

### 2.1. Cell Culture

Bone marrow was aspirated from 3 patients with critical limb ischemia of age group 65.3 ± 6.7 after receiving written informed consent and approval by the local ethical committee of the National Institute of Cardiovascular Diseases, Bratislava. This study was carried out by the Code of Ethics of the World Medical Association, Declaration of Helsinki (WMA Declaration of Helsinki, 2013). The local ethical committee of National Cardiovascular Institute, Bratislava, approved all of the experimental protocols, and the study was already published [15]. Bone marrow cells were isolated by density gradient centrifugation to obtain bone-marrow-rich product containing blood elements. Cells were plated in 75 flasks with low-glucose DMEM (Dulbecco’ modified Eagle medium, Sigma Aldrich, Germany) enriched with 10% fetal bovine serum (FBS), penicillin (100 IU/mL) and streptomycin (100 µg/mL), maintained at 37 °C in a humidified incubator with 5% CO_2_. After three days of culture, the medium and non-adherent cells were replaced, while adherent BM-MSCs were further grown. Medium change was performed to remove non-adherent cells at defined intervals. The MSCs were grown in medium up to passage 3 (P3) and were derived from 3 different donors.

For experiments, the 4th passage was transferred to a tissue culture dish in an amount of 0.5 × 10^6^ cells. Control cells were treated with cultivation medium (CTR) or 0.1% DMSO as a vehicle control (DMSO). Atorvastatin (Sigma, Schnelldorf, Germany) was dissolved in 0.1% DMSO to a final concentration of 10 µm in cultivation medium (ATO). Optimal concentration of atorvastatin (10 µm) was chosen based on literature search identifying in vitro experiments where pleiotropic effects of statin are claimed [16,17]. MSCs were pre-treated with 10 µm atorvastatin for 20 min (20 m ATO 10 µm), 2 h (2 h ATO 10 µm), 24 h (24 h ATO 10 µm), 48 h (48 h ATO 10 µm) or 96 h (96 h ATO 10 µm).

### 2.2. Cell Morphology, Viability and Characterization of BM-MSCs Expanded In Vitro

Cell morphology and characterization of BM-MSCs were evaluated before and after atorvastatin treatment. Imaging of BM-MSCs was performed using an M-795 inverted microscope (OPTIKA S.R.L., Ponteranica, Italy). Expression of surface antigens in BM-MSCs was quantified by flow cytometry (MACS Quant Analyzer, Miltenyi Biotec, Bergisch Gladbach, Germany; with MACSQuantify software 2.13.3) using the MSC Phenotyping kit (Miltenyi Biotec, Bergisch Gladbach, Germany) according to manufacturer’s instructions (≥95% of the MSC population must express CD73, CD90 and CD105, and these cells must lack expression (≤2%) of CD14, CD20, CD34 and CD45). Cell viability was assessed with propidium iodide (PI, Miltenyi Biotec, Bergisch Gladbach, Germany).

### 2.3. Immunofluorescence Staining

To investigate the morphological changes, BM-MSCs (1 × 10^5^ cells/dish) were seeded on Petri dishes and cultured as described above. After 48 h, cells were fixed with 4% paraformaldehyde and permeabilized with 0.2% Triton X/0.1% Tween/1 × PBS (30 m). The cells were then blocked with 2% goat serum (Sigma Aldrich, Schnelldorf, Germany) for 1 h and incubated with rabbit vimentin antibody (1:100 dilution; cat. No. D21H3 XP, Cell Signaling Technology, Beverly, MA, USA) for 1 h. After washing, the cells were incubated with the Alexa Fluor 488-conjugated Goat anti-mouse IgG (H + L) (1:500 dilution; Cell Signaling Technology, USA) for 1 h at room temperature. DAPI was used to stain cell nuclei. Fluorescent images were captured on a Ti-E microscope (Nikon instrument, Melville, NY, USA) at 10× magnification.

### 2.4. Gene Expression Determination

A quantitative polymerase chain reaction was performed to study the expression of specific genes of BM-MSCs, including *VEGF-A*, *HGF*, *IGF-1*, *IGF-2*, *IGF-1R*, *FGF-1R*, *AKT1*, *PI3KCA*, *mTOR*, *BAX*, *BCL-2*, and *HMGCR*.

Total RNA was isolated from BM-MSCs using the Tri-Reagent^®^ (Sigma Aldrich, Schnelldorf, Germany) according to the manufacturer’s instruction and phenol-chloroform extraction. Total RNA quantity was measured using the Qubit RNA XR Assay Kit (Thermo Fisher Scientific, Waltham, MA, USA). Reverse transcription was performed using the High-Capacity cDNA Reverse KIT with RNAse inhibitor (Thermo Fisher Scientific, Waltham, MA, USA). Quantification of mRNA expression was performed using SYBR Select Master Mix (Life Technologies, Waltham, MA, USA) on a StepOnePlus Real-Time PCR System (Life Technologies, Waltham, MA, USA) or TaqMan Universal PCR Master Mix kit on QuantStudio 5 Real-Time PCR System (Thermo Fisher Scientific, Waltham, MA, USA) according to the manufacturer’s instructions. Primers (Sigma-Aldrich, St.Louis, MO, USA) were designed to amplify human *VEGF-A*, *HGF*, *IGF-1*, *B2M*, *GAPDH* and *RPL13A* (Table 1). All primers were verified to produce a single PCR product with the correct molecular weight, and the absence of signal was verified when reverse transcription was omitted. The Pfaffl method was used to calculate the relative expression [18]. Results were normalized to the geometric mean of three most suitable reference genes (*RPL13A*, *B2M*, and *GAPDH*) [19]. Calculated normalized quantities were calibrated to the control group. The gene primer sequences are shown in Table 1, together with TaqMan assays utilized in this study.

### 2.5. Statistical Analysis

Statistical analysis was performed using GraphPad Prism version 5.00 (GraphPad Software, San Diego, CA, USA). At least three independent experiments performed in triplicate represent results expressed as mean ± standard deviation. The Shapiro–Wilk method was used for normality testing. Analysis of variance was performed, followed by Tukey’s multiple comparison test or t-test, where comparing two groups was appropriate. A *p*-value (*p) of <0.05 was considered as statistically significant.

## 3. Results

### 3.1. Characterization of BM-MSCs

**Figure 1:**
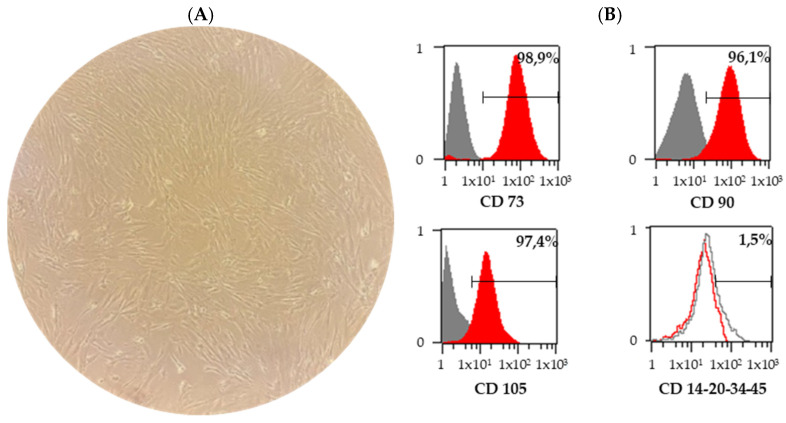
Morphology of human BM-MSCs and flow cytometry analysis of BM-MSCs expanded to passage three under normoxic conditions. (**A**) BM-MCSs exhibit an elongated fibroblast-like morphology (10× magnification). (**B**) BM-MSCs expressed characteristic mesenchymal stem cell markers (CD73, CD90, CD105) while the non-MSC markers (CD14, CD20, CD34 and CD45) were not detected. BM-MSC, bone-marrow-derived mesenchymal stem cell.

As shown in Figure 1A, human BM-MSCs at passage three were demonstrated to have an elongated, fibroblast-like morphology. To characterize BM-MSCs, we analyzed their surface markers using flow cytometry. The data revealed that BM-MSCs were uniformly positive for MSC markers CD73, CD90 and CD105, whereas they were negative for the hematopoietic progenitor cell marker CD34 and leukocyte common antigen CD45 (Figure 1B).

### 3.2. Effect of Atorvastatin Pre-Treatment on Characteristics and Viability of BM-MSCs

**Figure 2:**
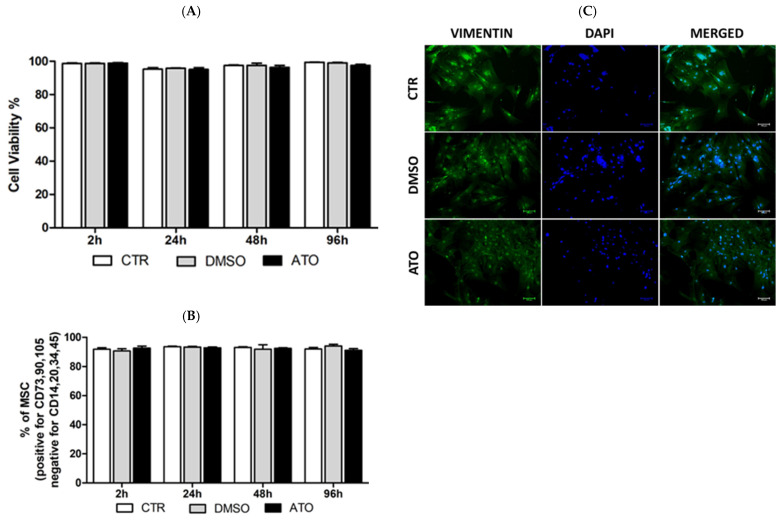
(**A**) Flow cytometry analysis of viability with propidium iodide staining in control (CTR), 0.1% DMSO and atorvastatin pre-treatment group (ATO) of BM-MSCs. Data are presented as the mean ± standard deviation (n = 3). (**B**) Quantifying cultured BM-MSCs by flow cytometry (expression of MSC-positive markers CD73, CD90 and CD105 and MSC-negative markers CD14, CD20, CD34 and CD45) in CTR, 0.1% DMSO and ATO group of BM-MSCs. Data are presented as the mean ± standard deviation (n = 3). (**C**) Fluorescence microscopy of BM-MSCs cultured with 0.1% DMSO and ATO after 48 h and stained for vimentin (green). Cell nuclei were counterstained with DAPI (blue) (scale bar 100 µm).

To provide insights into the mechanism of how atorvastatin regulates the viability and expression of MSCs markers, flow cytometry analysis was performed in four different time intervals of pre-treatment. We found that pharmacological stimulation with atorvastatin did not significantly affect cell viability in our experiment (Figure 2A). Viability averages in the control group were 98.6 ± 0.8, 95.2 ± 1.5, 97.5 ± 0.6 and 99.4 ± 0.2% in 2 h, 24 h, 48 h and 96 h intervals. The relative values of viability after 2 h, 24 h, 48 h and 96 h of atorvastatin pre-treatment were 98.9 ± 0.5, 95.0 ± 2.1, 96.3 ± 2.1 and 97.5 ± 1.0%. The expression of MSC surface markers was not changed by atorvastatin stimulation (Figure 2B). The immunofluorescent staining of total nuclei with DAPI and intermediate cytoskeletal filament with anti-vimentin antibodies showed no significant differences between untreated control BM-MSCs, DMSO and atorvastatin-treated BM-MSCs (Figure 2C).

### 3.3. Atorvastatin’s Effect on Gene Expression in BM-MSCs

**Figure 3:**
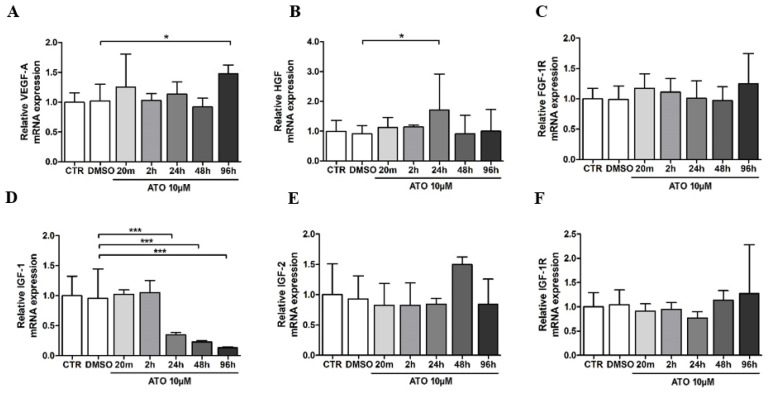
Effect of atorvastatin on (**A**) *VEGF-A*, (**B**) *HGF*, (**C**) *FGF-1R*, (**D**) *IGF-1*, (**E**) *IGF-2* and (**F**) *IGF-1R* mRNA expression in BM-MSCs. Data are presented as mean ± standard deviation of three experiments from different donors (n = 3) performed in triplicate (* *p* < 0.05; *** *p* < 0.001 compared to 0.1% DMSO group).

We evaluated the effect of atorvastatin on VEGF-A production in BM-MSCs. First, we performed real-time RT-PCR to determine the *VEGF-A* mRNA level in atorvastatin-treated BM-MSCs. Compared with the control group, DMSO did not alter the mRNA level of *VEGF-A* (102.3 ± 28%). Atorvastatin treatment significantly increased the *VEGF-A* mRNA level to 148.1 ± 14% at the 96 h time point when compared to the DMSO-treated group (Figure 3A). Since the pleiotropic effect of atorvastatin involves many effectors, we investigated its action on growth factors and receptors working through the PI3K/AKT signaling pathway. Treatment with atorvastatin for 24 h significantly upregulated *HGF* mRNA expression in BM-MSCs (171.4 ± 120%) compared to the DMSO group (90.8 ± 27.5%). In contrast, *FGF-1R* mRNA expression remained unaffected at all time points compared to the DMSO group (Figure 3B,C). Atorvastatin pre-treatment significantly decreased the mRNA expression level of *IGF-1* after 24 h (34.7 ± 4.3%), 48 h (23 ± 2.3%) and 96 h (13.3 ± 1.3%) compared to DMSO treatment (96 ± 48%) (Figure 3D). The gene expression of *IGF-2* was increased in 48 h of atorvastatin pre-treatment (149.8 ± 12.3%) compared with DMSO treatment (93.3 ± 37.8%). However, it was not statistically significant (Figure 3E). The downregulation of *IGF-1R* mRNA expression was observed at a 24 h time interval of atorvastatin pre-treatment (77.1 ± 13%) compared to the DMSO group (104.6 ± 30.1%). Still, this change was not significant, as in none of the time intervals (Figure 3F).

**Figure 4:**
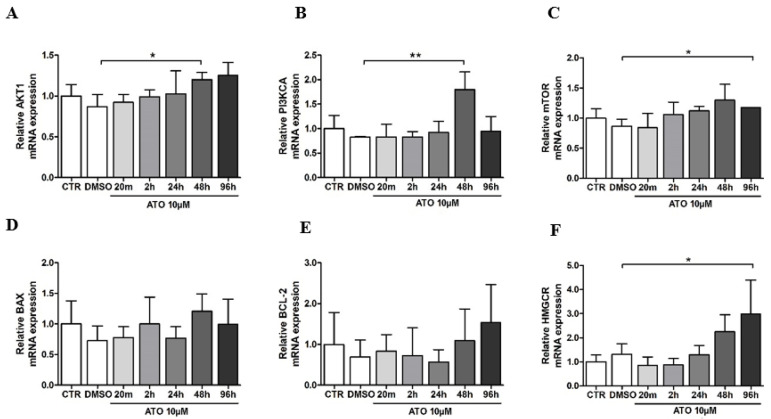
Effect of atorvastatin on (**A**) *AKT1*, (**B**) *PI3KCA*, (**C**) *mTOR*, (**D**) *BAX*, (**E**) *BCL-2* and (**F**) *HMGCR* mRNA expression in BM-MSCs. Data are presented as the mean ± standard deviation of three experiments from different donors (n = 3) performed in triplicate (* *p* < 0.05; ** *p* < 0.01 compared to 0.1% DMSO group).

The PI3K/AKT pathway is one of the most important signaling pathways responsible for the endothelial function, regulating the vascular tone and angiogenesis [20]. Therefore, we determined whether atorvastatin affects *PI3K* and *AKT* gene expression in BM-MSCs. As shown in Figure 4A, the RT-PCR analysis revealed an upregulation of *AKT1* mRNA to 120.1 ± 8.8% compared with the DMSO group (86.8 ± 15.1%) at 48 h pre-treatment. Compared to the DMSO group (82.4 ± 1.5%), atorvastatin treatment significantly increased *PI3KCA* mRNA in BM-MSCs to 179.9 ± 35.7% at 48 h (Figure 4B). Together, these results indicate that the PI3K/AKT pathway is modulated on the mRNA level in atorvastatin-treated BM-MSCs.

An important downstream target of PI3K/AKT is the serine/threonine kinase, the mammalian target of rapamycin (mTOR), which, via signaling, is involved in the control of cell growth and proliferation. We performed an RT-PCR analysis to determine the *mTOR* mRNA expression in atorvastatin-treated BM-MSCs. The *mTOR* mRNA expression was significantly increased in BM-MSCs after 96 h of treatment with atorvastatin (117.8 ± 0.07%) compared to the DMSO group (86.7 ± 11.7%) (Figure 4C). After pharmacological treatment, the expression of pro- and anti-apoptotic genes, BAX, and BCL-2 was investigated in the bone marrow mesenchymal stem cell population. In our study, *BAX* and *BCL-2* mRNA expression were not significantly changed after atorvastatin pre-treatment (Figure 4D,E). 3-hydroxy-3-methylglutaryl coenzyme A reductase (*HMGCR*), a gene associated with atorvastatin’s effect, was significantly upregulated in the 96 h time interval (299.5 ± 139.8%) compared to the DMSO group (132.1 ± 42.2%) (Figure 4F).

## 4. Discussion

Stem cell transplantation represents a unique therapeutic tool for tissue engineering and regenerative medicine. Many studies have already compared different types of stem cells and their dose, delivery route and timing based on preclinical and clinical findings [21]. However, in order to improve the therapeutic efficacy of bone-marrow-derived mesenchymal stem cells (BM-MSCs), a more comprehensive understanding of in vitro culture parameters that enhance their paracrine capabilities during expansion is required. Multiple studies indicate that combining statins and stem cell transplantation might improve stem cell therapeutic efficacy in regenerative medicine [11,13]. The elucidation of the pharmacological actions of 3-hydroxy-3-methylglutaryl coenzyme A (HMG-CoA) reductase inhibitors resulted in the realization that the benefit of these agents exceeds lowering cholesterol levels. These properties involve favorable effects on the endothelial function, ability to stabilize atheromatous plaques and impact on smooth muscle proliferation, as well as having an antithrombic effect and stimulating the fibrinolytic mechanism with an improvement in blood viscosity and decreased LDL oxidation. Atorvastatin is a synthetic HMG-CoA reductase inhibitor that is well tolerated and has been investigated in long-term clinical trials. Generally, atorvastatin is more effective at lowering serum LDL cholesterol, total cholesterol and levels of triglyceride than equivalent doses of simvastatin, lovastatin, fluvastatin or pravastatin [22,23]. Atorvastatin is one of the most frequently studied statins, with the effect of increasing the survival of implanted stem cells in animal models [5,11,24,25]. The objective of the present study was to evaluate the effect of pre-treatment with atorvastatin on the characteristics and properties of BM-MSCs, mainly if atorvastatin affected BM-MSCs gene expression.

Many previous experimental and clinical studies suggest different or even opposite outcomes of statins’ pre-treatment on the MSCs viability. While some authors found that statins promote the viability of adipose-tissue-derived MSCs transplanted into infarcted hearts [26], other studies demonstrated how atorvastatin and simvastatin progressively reduced the viability of MSCs [27]. To clarify the effect of atorvastatin on the survival of BM-MSCs, we assessed cell viability with propidium iodide using flow cytometry. Our results demonstrate that atorvastatin did not affect the viability of BM-MSCs in any of the time intervals of pre-treatment. To verify their MSCs phenotype and record any changes through the pre-treatment, cells were characterized by flow cytometry. They were positive for markers of mesenchymal stem cells, namely CD73, CD90 and CD105, and did not express markers typical for hematopoietic and endothelial cells, CD14, CD20, CD34 and CD45 [28]. Our results did not record any significant alterations in the expression of mentioned markers during atorvastatin pre-treatment.

Although there are numerous questions about the precise mechanisms underlying the therapeutic effects associated with engrafted stem cells, a large amount of evidence suggests that paracrine mechanisms mediated by MSCs may play an essential role in tissue regeneration [29,30,31,32,33]. In the present study, atorvastatin significantly increased the transcription level of *VEGF-A* and the transcription of *HGF*. VEGF-A binds to tyrosine kinase receptors on endothelial cells and activates AKT phosphorylation with a unique effect on angiogenesis. Some consequences of AKT phosphorylation include MSC differentiation into endothelial progenitor cells and the activation of the PI3K/AKT-dependent signaling pathway involved in vasculogenesis [34]. Amongst factors participating in angiogenesis, VEGF is the most relevant since it modulates the function of vascular and non-vascular cells and promotes every step of angiogenesis [35,36]. Following our results, other studies showed the ability of statins, specifically simvastatin, to upregulate VEGF expression in MSCs [37,38]. The authors also found that rosuvastatin and atorvastatin increased the expression of FGF, IGF-1 and HGF. The treatment of MSCs with statins demonstrated an increased capillary density in a rat model of coronary microembolism and in in vitro tube formation assays [39,40]. After rosuvastatin treatment, a capillary-like tube formation was observed in co-cultured human umbilical vein endothelial cells with MSCs. Rosuvastatin-induced MSCs secreted angiogenic growth factors and increased VEGF, HGF and platelet-derived growth factor expression [34]. The expression analysis of *IGF-1* and *IGF-2* in our study revealed an opposite influence of atorvastatin. The expression of *IGF-1* was significantly reduced after atorvastatin pre-treatment, whereas *IGF-2* had an increased tendency and the expression of *IGF-1R* was variable without significant alterations. The report by Mieno et al. documented that IGF-1 enhances the migratory response of MSCs to the stromal cell-derived factor-1α, which is a potent stem cell chemoattractant and plays an important role in the modulation of stem cell functions through the activation of molecular pathways of cell growth, proliferation and survival [41]. Longobardi et al. found that IGF-1 supports the differentiation of human BM-MSCs into chondrocytes and is the most abundant growth factor in the bone matrix [42]. Moreover, the receptor for IGF-1 is involved in stem cells’ pluripotent or multipotent properties. Several investigations reported the involvement of IGF-1R signaling in maintaining stem cell characteristics and enhancing stem cell therapy efficacy [43]. Our data obtained from atorvastatin pre-treatment did not demonstrate significant alterations in the relative mRNA expression of *IGF-1R*, which may be associated with the stable maintenance of stem cell characteristics.

It is necessary to provide insight into the signaling pathway by which atorvastatin regulates the paracrine function of MSCs, clarifying how angiogenic factors alter their downstream targets. The PI3K/AKT signaling pathway is well-known for numerous cellular functions, including proliferation, migration, survival, metabolism and angiogenesis [20]. A range of molecules including insulin, glucose, many growth factors and cytokines initiate PI3K/AKT signaling via the activation of receptor tyrosine kinases (RTK) and G-protein-coupled receptors. Activated PI3K converts phosphatidylinositol-4, 5-bisphosphate into phosphatidylinositol-3, 4, 5-trisphosphate and further activates downstream effectors such as AKT and the mammalian target of rapamycin (mTOR) [44]. PI3K/AKT/mTOR is a substantial and complex signaling pathway that functions in many cellular processes essential for homeostasis, including the cell cycle, survival, inflammation, metabolism and apoptosis [45]. Our findings support previous studies’ results showing that statin treatment is linked to the PI3K/AKT pathway [34]. Our results indicate that the PI3K/AKT signaling pathway was modulated by atorvastatin by an increased mRNA expression of *PI3K* and *AKT* after 48 h pre-treatment. Moreover, our data showed a significant upregulation of *mTOR* transcription, suggesting that the mechanism of the atorvastatin effect on BM-MSCs may be mediated through the PI3K/AKT/mTOR pathway.

Furthermore, the PI3K/AKT pathway is essential in regulating Bcl-2 family members, which control the mitochondrial membrane integrity and release cytochrome c from mitochondria during apoptosis [46]. The Bcl-2 family involves both pro- and anti-apoptotic members with up to four conserved Bcl-2 homology domains. Previous studies demonstrated the important role of statins in regulating Bcl-2 family members. Yang et al. found that simvastatin treatment improved the therapeutic efficacy of MSCs transplanted into infarcted swine hearts, which was demonstrated by inhibiting the pro-apoptotic protein Bax and increasing the anti-apoptotic protein Bcl-2 [47]. Similar findings were published by other authors, who showed that rosuvastatin decreased the levels of pro-apoptotic proteins Bim and Bax and increased the anti-apoptotic proteins Bcl-xL and Bcl-2 [26]. However, our data obtained from atorvastatin-treated BM-MSCs showed no significant differences in the mRNA expressions of *BAX* and *BCL-2*.

One supposed mechanism of atorvastatin-induced regulation could be mediated by microRNAs (miR), a family of noncoding single-stranded RNAs consisting of 22 nucleotides regulating gene expression in post-transcriptional mechanism [48]. They modulate multiple cellular processes involving proliferation, differentiation and migration. An example is miR-1 and miR-133, key regulators of IGF-1 and IGF-1R. These miRNAs negatively regulate the IGF-1/AKT signaling pathway by targeting its positive regulators, IGF-1 and IGF-1R [49]. Another example of miR involvement in MSCs physiology was already studied in the work by Li et al. The authors focused on the mechanism by which atorvastatin pre-treatment enhances MSC migration. CXC chemokine 4 (CXCR4) signaling plays a central role in stem cell migration and is regulated by miR-146a. They found that atorvastatin pre-treatment in vitro upregulated CXCR4 and induced MSCs migration by suppressing miR-146a. Thus, the miR-146a/CXCR4 signaling pathway contributes to MSCs migration and homing induced by atorvastatin pretreatment [50].

Another perspective demonstrates MSC-derived exosome approaches, which hold great promise as a potential novel cell-free therapy for cardiac repair [51]. A study by Huang et al. investigated the pro-cardioprotective effect of atorvastatin on MSC-derived exosomes. Exosomes were isolated from control MSCs and atorvastatin-pre-treated MSCs. They were delivered to endothelial cells and cardiomyocytes in vitro under hypoxia and serum deprivation conditions or in vivo in an acutely infarcted Sprague-Dawley rat heart. They identified long non-coding RNA H19 as a mediator of the role of the atorvastatin-pre-treated MSCs exosome in regulating the expression of miR-675 and activation of proangiogenic factor VEGF and intercellular adhesion molecule-1. They concluded that atorvastatin pre-treatment promotes the function of MSC-derived exosomes in enhancing angiogenesis, protecting cardiomyocytes and improving cardiac function after infarction, thus potentially leading to a prospective strategy for the improvement of therapeutic outcomes [52].

## 5. Conclusions

In summary, in our in vitro study, the atorvastatin pre-treatment of BM-MSC did not affect the cell morphology, viability or expression of MSC markers. Our data demonstrated a significant increase in the mRNA expression of angiogenic factors, *VEGF-A* and *HGF* in atorvastatin-treated BM-MSCs. In contrast, there was a significant reduction in *IGF-1* transcripts. Moreover, the results implicate atorvastatin’s role in the PI3K/AKT signaling pathway with the upregulation of *mTOR* transcription. We propose that atorvastatin benefits BM-MSC treatment for its ability to upregulate angiogenesis-related genes expression and transcripts of the PI3K/AKT/mTOR pathway. However, other more extensive studies are needed.

## Figures and Tables

**Table 1:** Primer sequences and Primer ID used for the qRT-PCR analysis of selected gene expression.

Gene	Sense: 5′	Anti-Sense: 5′	
*VEGF-A*	GGTCCCAGGCTGCACCCATG	ATTGCAGCAGCCCCCGCATC	
*HGF*	CTTCCATTCACTTGCAAGGCT	TGTTCCCTTGTAGCTGCGTC	
*IGF-1*	TGGATGCTCTTCAGTTCGTG	ATCCACGATGCCTGTCTGA	
*B2M*	TCCGTGGCCTTAGCTGTGCT	TCCATTCTCTGCTGGATGACGTGAG	
*GAPDH*	TCCTGTTCGACAGTCAGCCGC	CATGGTGTCTGAGCGATGTGGC	
*RPL13A*	CTTTTCCAAGCGGCTGCCGAAGA	GGCCTCGACCATCAAGCACCAG	
**Gene**	**Primer ID**	**Gene**	**Primer ID**
*FGF-1R*	Hs00241111	*BAX*	Hs00180269
*IGF-2*	Hs01005963	*BCL-2*	Hs00608023
*IGF-1R*	Hs00609566	*HMGCR*	Hs00168352
*PI3KCA*	Hs00907957	*B2M*	Hs99999907
*AKT1*	Hs00178289	*GAPDH*	Hs99999905
*mTOR*	Hs00234508	*RPL13A*	Hs01578912

## Data Availability

The data that support the findings of this study are available upon request.
